# Metal‐Organic Frameworks‐Based Copper Catalysts for CO_2_ Electroreduction Toward Multicarbon Products

**DOI:** 10.1002/EXP.70011

**Published:** 2025-02-12

**Authors:** Chen Qin, Xuheng Li, Ting Wang, Zhen Xu, Kai‐Jie Chen, Fuping Pan

**Affiliations:** ^1^ School of Chemistry and Chemical Engineering Northwestern Polytechnical University Xi'an Shaanxi China; ^2^ Chongqing Innovation Center Northwestern Polytechnical University Chongqing China

**Keywords:** catalyst design, C−C coupling, CO_2_ reduction, copper, metal‐organic frameworks

## Abstract

Copper (Cu) is the most promising catalyst for electrochemical CO_2_‐to‐C_2+_ conversion, whereas performance remains below practical thresholds due to the high energy barrier of C−C coupling and lack of effective approaches to steer the reaction pathway. Recent advances show that metal‐organic frameworks (MOF) could be a promising platform as support, pre‐catalyst, and co‐catalyst to modify the electronic structure and local reaction environment of Cu catalysts for promoting CO_2_‐to‐C_2+_ reduction by virtue of their great tunability over compositions and pore architectures. In this review, we discussed general design principles, catalytic mechanisms, and performance achievements of MOF‐based Cu catalysts, aiming to boost catalyst refinement for steering CO_2_ reduction pathway to C_2+_ products. The fundamentals and challenges of CO_2_‐to‐C_2+_ reduction are first introduced. Then, we summarized design conceptions of MOF‐based Cu catalysts from three aspects: engineering the electronic properties of Cu, regulating the local reaction environment, and managing site exposure and mass transport. Further, the latest progress of CO_2_ reduction to C_2+_ products over MOF‐based Cu catalysts, namely Cu‐based MOF, MOF‐derived Cu, and Cu@MOF hybrid catalysts, are discussed. Finally, future research opportunities and strategies are suggested to innovate the rational design of advanced MOF‐based Cu catalysts for electrifying CO_2_‐to‐C_2+_ transformation.

## Graphical abstract

This review discusses design principles, preparation approaches, and performance achievements of metal‐organic frameworks (MOF)‐based Cu catalysts for electrochemical CO_2_ reduction to C_2+_ products. The correlation between synthesis, composition/structure, and catalytic properties was critically discussed to reveal catalytic mechanisms and performance‐determining factors. The strategies are suggested to address the remaining challenges for designing advanced MOF‐based Cu catalysts for CO_2_‐to‐C_2+_ transformation.

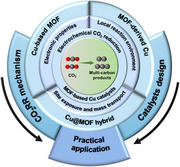

## 1 Introduction

The extensive use of fossil fuels leads to ever‐increasing CO_2_ levels in the atmosphere, causing serious environmental problems such as global warming, glacier melting, and biological loss. Up to 2023, the global average concentration of atmospheric CO_2_ has reached a record high of 423 ppm, which may reach 570 ppm by 2100 according to the Intergovernmental Panel on Climate Change [1]. It is urgent to develop sustainable technologies for converting CO_2_ into valuable products to meet global carbon neutrality. In recent years, several CO_2_ conversion technologies have been developed, including electrocatalysis [2, 3, 4, 5], photocatalysis [6, 7], thermocatalysis [8, 9], biocatalysis [10]. Among them, electrocatalytic CO_2_ conversion is considered the most promising method as it can be powered by renewable electricity and operated under room temperature and ambient pressure as well as has high CO_2_ conversion efficiency, being able to improve commercial operability and reducing cost.

The CO_2_ reduction reaction (CO_2_RR) is the key process in CO_2_ electrolysis. The main products from CO_2_RR includes C_1_ (CO, CH_4_, HCOOH, etc.) and C_2+_ products (C_2_H_4_, C_2_H_5_OH, CH_3_COOH, C_3_H_7_OH, etc.). Because of the high economic benefits and large market need, C_2+_ products are more favored than C_1_ [11]. However, there are a series of challenges in reducing CO_2_ to C_2+_ products, including high overpotential, poor selectivity, and low current density, which are mainly caused by the sluggish reaction kinetics of C−C coupling, complex catalytic mechanisms, and uncontrollable reaction pathways [12, 13, 14, 15]. In addition, CO_2_RR is always accompanied by competitive hydrogen evolution reactions (HER) due to their close thermodynamical equilibrium potentials, which greatly limits the current efficiency. Moreover, according to the linear adsorption relationship, if the intermediate adsorption on a catalyst is too strong, product desorption will be very difficult, and vice versa. Thus, it is difficult to continue the proton‐coupled electron transfer (PCET) process to produce C_2+_ products, and the selectivity of a single C_2+_ product is poor.

The electrocatalyst is the most important factor in determining the efficiency, selectivity, and stability of CO_2_ reduction [16]. Different catalysts show different selectivity in CO_2_ reduction [17]. For example, Au, Pd, Ag, and other first‐line transition metal catalysts produce CO as the main product; Sn, In, and Pb mainly generate HCOO^−^ [18]. Cu is the only metal with a negative adsorption energy for *CO and a positive adsorption energy for *H, which thus suppresses HER and favors CO_2_ reduction [19]. More importantly, the *CO binding energy on Cu is moderate, which is conducive to C−C coupling to generate C_2+_ products [20]. Although recently developed Cu‐free catalysts (such as NiO, FeP, heteroatom‐doped carbon, etc.) have shown the ability to reduce CO_2_ to C_2+_ products, their performance remains much lower than Cu catalysts [21, 22]. So far, Cu is the most promising catalyst in practically converting CO_2_ to C_2+_ products. However, the performance of Cu‐based catalysts is still below the requirement for large‐scale applications in industry [23]. More challengingly, due to the limitations of in situ characterization methods and theoretical calculations, the mechanisms of C−C coupling, the most key step in generating C_2+_ products, is still not fully understood, limiting the precise design and synthesis of Cu electrocatalysts.

**FIGURE 1:**
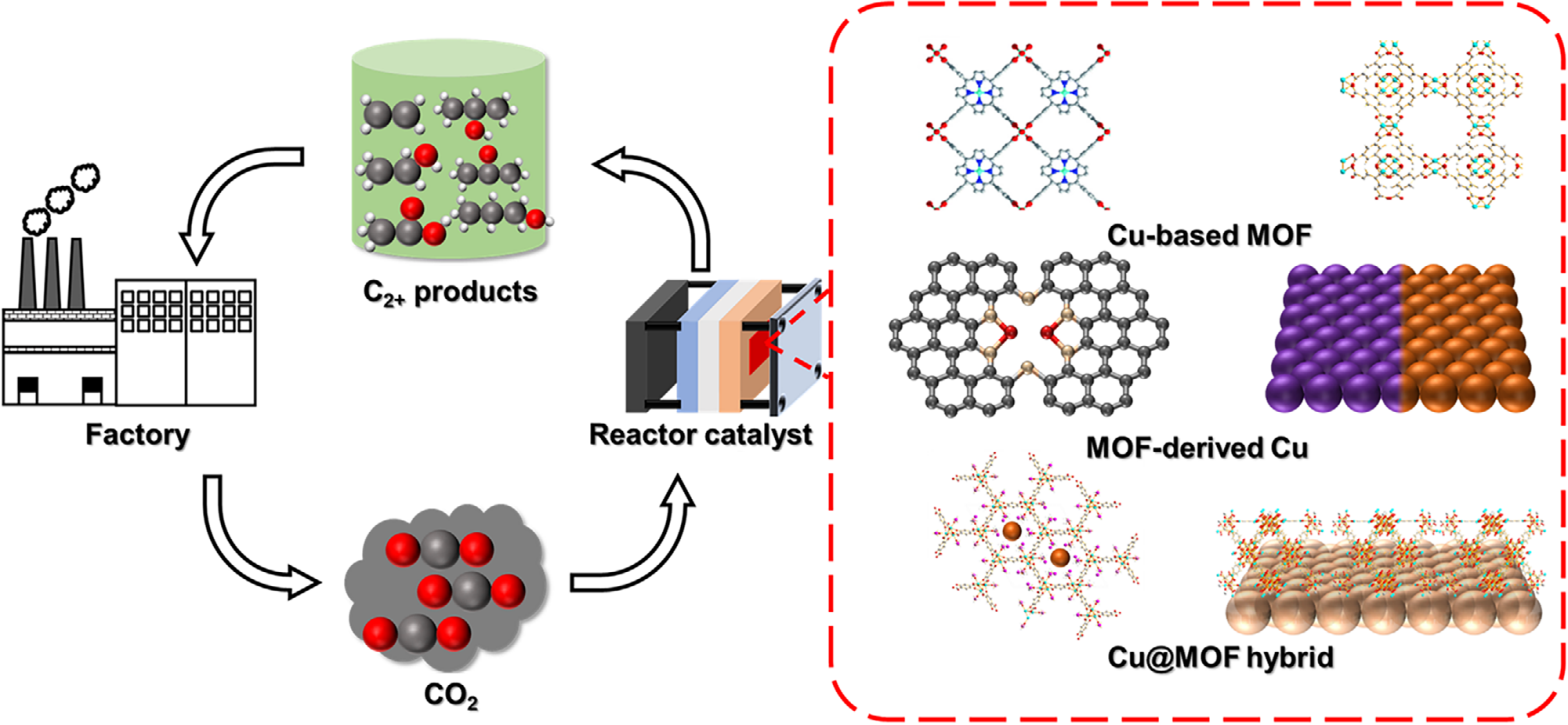
MOF‐based Cu catalysts for CO_2_ electroreduction to C_2+_ products.

The key factors toward CO_2_‐to‐C_2+_ reduction include the adsorption strength of vital intermediates, the appropriate distance between catalytic sites, exposure of surface sites, electron transfer, and mass transport of CO_2_/intermediates. All of those factors can influence C−C coupling, reaction path, and catalytic rate thus determining the distribution and generation rate of final products. Due to the high surface area, high porosity, adjustable pore structure, great structure designability, and easy modification [24, 25, 26, 27, 28, 29, 30]. MOF is emerging as a promising platform to effectively regulate CO_2_RR behaviors of Cu‐based electrocatalysts to produce C_2+_ products. The design strategies of using MOF to promote CO_2_ reduction of Cu sites can be classified into the following three ways (Figure 1). Firstly, Cu‐based MOF can be directly used as the catalyst. The highly ordered framework of MOF helps prevent the agglomeration deactivation of Cu sites and improves the utilization efficiency of atoms [31]. The large surface area and high porosity are conducive to the exposure of active sites [32] and the mass transport of CO_2_ to the catalytic site [33]. Moreover, due to the structural designability of MOF, it is expected to achieve precise regulation of C−C coupling and improve the selectivity of specific products by changing ligands, introducing counter anions, and regulating the coordination microenvironment of Cu sites. The introduction of other metals in the metal cluster of MOF to construct double metal sites is also a productive strategy to promote C−C coupling by designing Cu−M sites with appropriate distances [34]. Secondly, through ex situ pyrolysis and in situ electrochemical reconstruction, MOF can be used as the precursor to prepare Cu‐based catalysts with enhanced control in adjusting the compositions and exposing specific crystal faces of MOF‐derived Cu catalysts. Finally, combining Cu with MOF can produce Cu@MOF hybrid catalysts [35]. For instance, Cu can be loaded into the porous channel of MOF. Because of the spatial confinement of the MOF's channel, a high concentration of *CO could be formed on the surface of Cu, promoting C−C coupling via *CO dimerization [36]. Despite great achievements on MOF‐based Cu catalysts, there is still a big gap between the current CO_2_‐to‐C_2+_ performance and the practical application in the industry. The poor performance is mainly caused by a lack of general catalyst design principles, a poor understanding of catalytic mechanisms, and a lack of effective methods to fabricate MOF‐based Cu catalysts.

In this review, the recent progress on using MOF‐based Cu catalysts for CO_2_‐to‐C_2+_ reduction was insightfully reviewed to clarify catalyst design, working mechanisms, and catalyst preparation. Firstly, the fundamentals and challenges faced by CO_2_‐to‐C_2+_ reduction were summarized. Secondly, the design principles of using MOF to promote catalytic properties of Cu were outlined, including regulation of the electronic structure of Cu sites (crystal surface, alloy, coordination, metal‐support interaction), tuning local reaction environment (local pH, ions in electrolyte, enrichment of reactive intermediates), and enhancement in site exposure and mass transport. Then, we summarized the preparation and application of three kinds of MOF‐based Cu catalysts for CO_2_‐to‐C_2+_ conversion, namely Cu‐based MOF, MOF‐derived Cu, and Cu@MOF composites. Finally, we proposed prospective and strategies for the future development of high‐performance MOF‐based Cu catalysts to propel practically sustainable CO_2_‐to‐C_2+_ conversion.

## 2 Fundamentals and Challenges in CO_2_‐to‐C_2+_ Reduction

The CO_2_RR process typically undergoes more complexly than other typical electrochemical reactions, such as hydrogen and oxygen electrocatalysis. Previous studies show that CO_2_RR involves various processes of 2, 4, 6, or even more electrons and protons transfer, which can produce various products depending on different electrocatalysts or reaction conditions. In this section, we will discuss the basic mechanism of CO_2_ reduction and the challenges faced by MOF‐based Cu catalysts.

### 2.1 CO_2_ Activation

**FIGURE 2:**
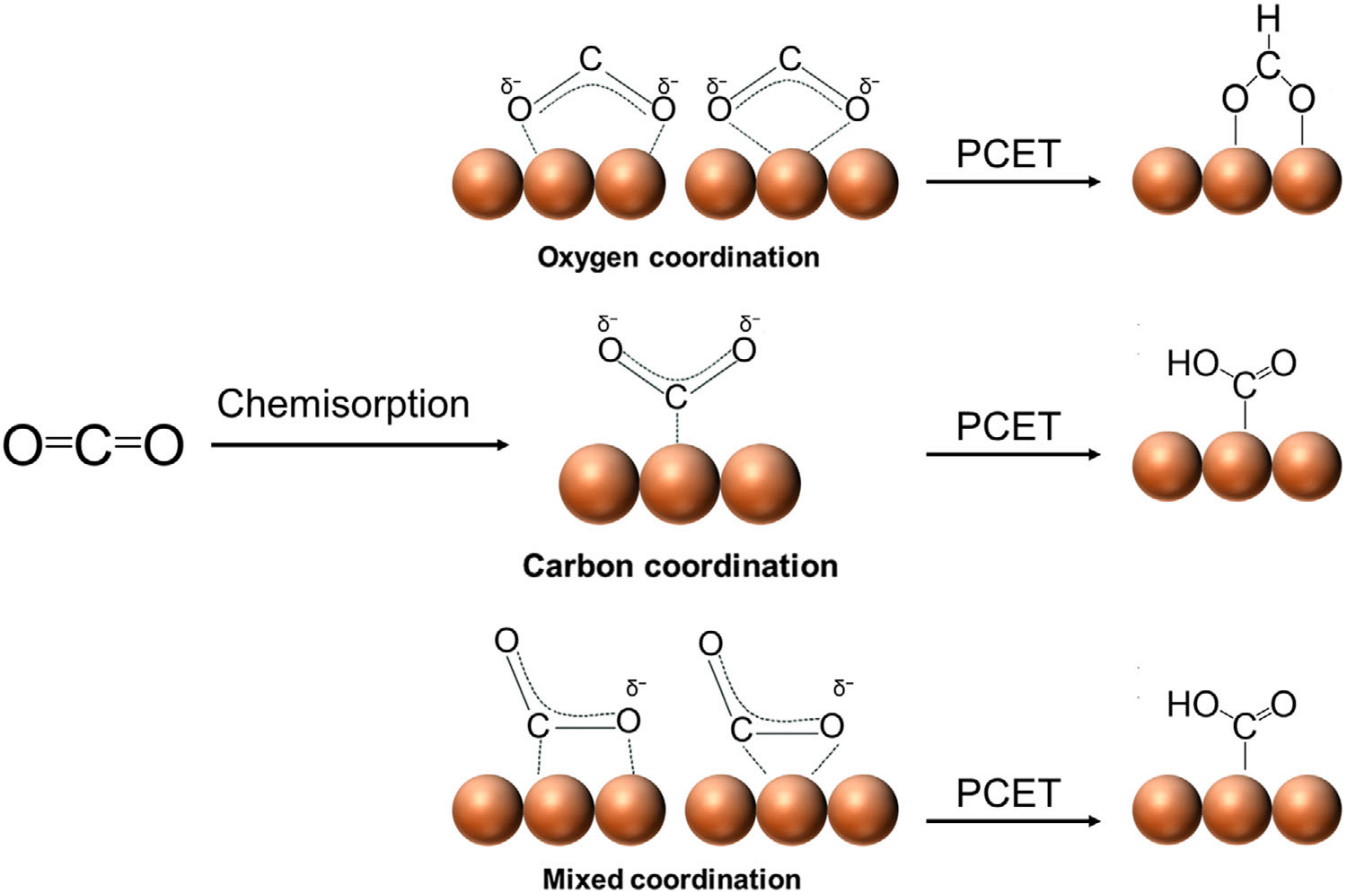
Proposed mechanisms for CO_2_ activation.

CO_2_ is a highly stable molecule under ambient conditions due to its strong chemical bonds and linear geometry (the dissociation energy of C═O bonds is as high as 750 KJ mol^−1^), which makes it hard for CO_2_ to be activated, resulting in low reaction activity [37]. Therefore, it is indispensable to weaken the strength of the C═O bond of CO_2_ and reduce the high energy barrier required for CO_2_ reduction by transforming the linear structure of CO_2_ into a bent configuration. Generally, three processes have been proposed to describe the mechanism of CO_2_ activation (Figure 2). One activation process is the adsorption of CO_2_ on the active site of an electrocatalyst via the bonding of the C atom of CO_2_ with the surface site to form a *CO_2_^δ−^ species [38]. Further, the *CO_2_^δ−^ species undergoes a PCET process to generate C‐bond *COOH. Alternatively, activation processes via O coordination and mixed C/O coordination have also been proposed. The *COOH is the intermediate to produce *CO upon the breakage of the C═O bond and *CO‐derived deep‐reduced products, while the *OCOH is the intermediate for the generation of HCOOH without the breakage of the C═O bond [39]. The equilibrium potential for PCET‐enabled transformation of O‐coordinated and mix‐coordinated *CO_2_^δ−^ is more positive than the one required for the reduction of C‐coordinated *CO_2_^δ−^, thus being proposed as favorable activation pathways [38].

### 2.2 Reaction Pathways

A typical CO_2_ reduction process begins with CO_2_ activation, which is the first crossroad of different products. The resulting *CO_2_^δ−^ intermediate will then go through different reaction pathways via the stepwise coupling with H^+^ and/or e^−^ to form different intermediates of C_1_ or C_2+_ products [40, 41], in which the product classification mainly depends on the adsorption state, binding strength, and scaling relationship of the involved intermediates.

**FIGURE 3:**
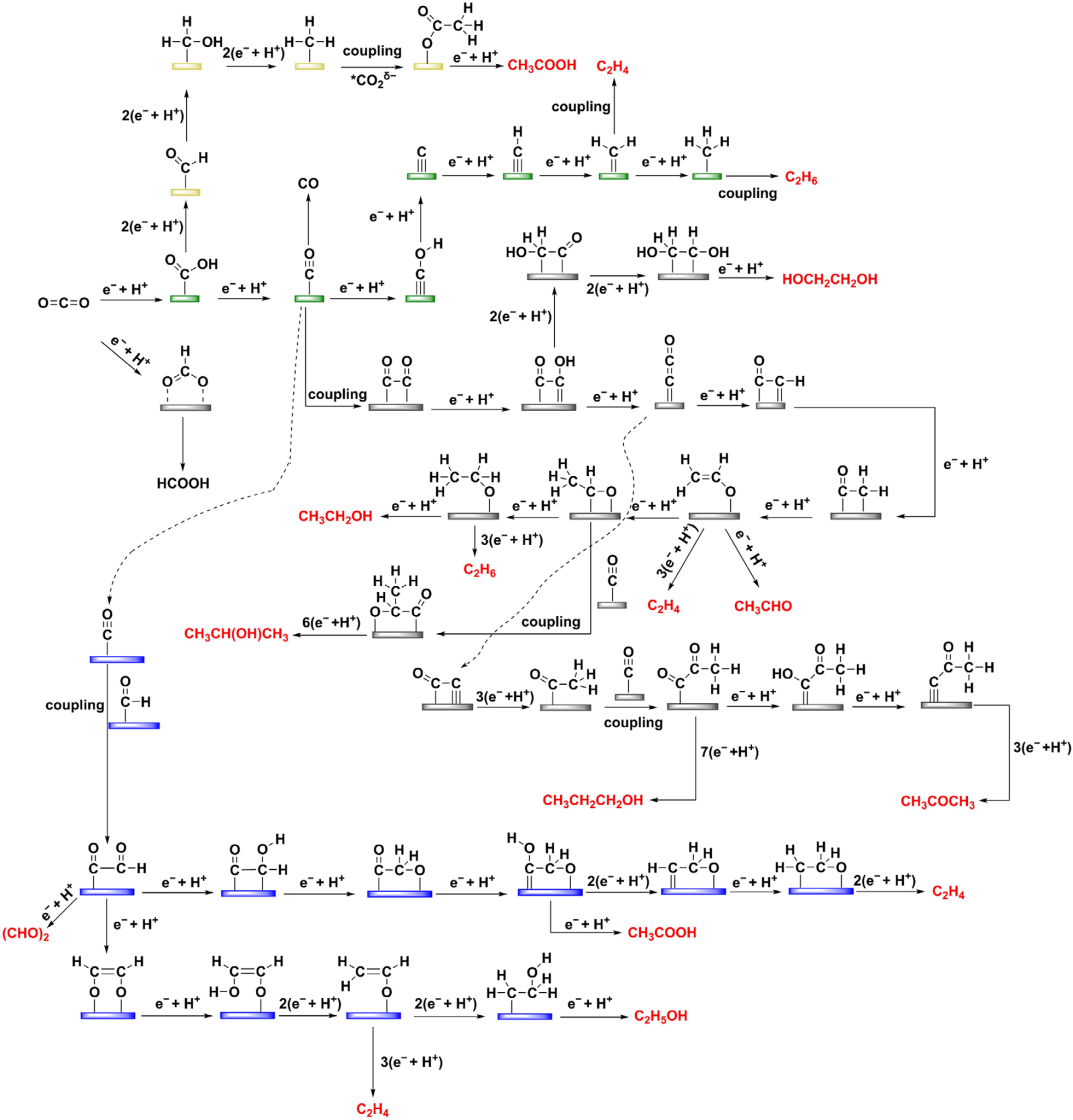
Overview of CO_2_RR pathways toward different C_2+_ products.

In general, the reduction of CO_2_ to O‐bonded *OCOH is the path to produce HCOOH [42, 43]. Instead, the reduction of CO_2_ to C‐bonded *COOH will produce a more important *CO intermediate, which is an intersection where deep‐reduced C_1_ and C_2+_ products are produced [44, 45, 46, 47]. When *CO is desorbed directly from the active site, gaseous CO is produced. Alternatively, *CO can trigger a series of PCET processes, accompanied by the cleavage of the C═O bond and the formation of the C−H bond. New C_1_ intermediates such as *CHO and *COH are then formed and eventually converted to CH_4_ and CH_3_OH [48, 49]. When *CO does not desorb from the active site, the C−C coupling with other C_1_ intermediates (such as *CHO, *COH, and *CH_x_) is possible, being able to produce C_2+_ products. In this Review, the mechanisms of CO_2_ reduction to form C_2+_ products were classified into four coupling pathways, including *CO−*CO, *CO−*CHO, *CH_x_−*CO_2_, and *CH_x_−*CH_x_ (Figure 3). The preference for these paths depends on many factors, such as the applied voltage, electrolyte environment, atomic‐level structure of the active site, etc.

In the *CO‐*CO path (Figure 3, gray path), it has been confirmed by experiments and theoretical calculations that the *CO dimerization is the rate‐determining step (RDS) [50], and the formation rate of the C—C bond is proportional to the surface coverage of *CO. C_2_H_4_ and C_2_H_5_OH are the most common products in the *CO−*CO pathway. The *C═CO is the key intermediate after *CO−*CO, which is further transformed into *OCH═CH_2_. The *OCH═CH_2_ reduction is the crossroad to C_2_H_4_ or C_2_H_5_OH [51]. In addition, *OCH═CH_2_ may also produce CH_3_CHO, but this intermediate cannot be easily desorbed from the catalyst surface, resulting in a further reduction to form C_2_H_5_OH. Alternatively, *CO is preferentially hydrogenated at more negative potentials to give *CHO, and *CO−*CHO coupling can occur (Figure 3, purple path) [52]. This path can also generate common C_2+_ products. There are another two C−C coupling pathways, as represented by green and yellow routes, which are less popular than the gray and blue paths. The green path occurs via *CH_x_−*CH_x_ coupling, mainly yielding C_2_H_4_ and C_2_H_6_ [53]. The yellow path undergoes the coupling of *CH_3_ with adsorbed *CO_2_ forming *OCOCH_3_ and producing CH_3_COOH, which happens at high overpotentials due to the high energy barrier of the *CH_x_−*CO_2_ coupling [54].

Compared to C_2_ products, C_3_ products undergo more C−C coupling and PCET steps, and the mechanism is more complex [55, 56]. For CH_3_COCH_3_ and CH_3_CH_2_CH_2_OH, it is proposed that the second C−C bond formation occurs via *COCH_3_ and *CO coupling. The formed *COCOCH_3_ further undergo three‐step and seven‐step PCET processes to produce CH_3_COCH_3_ and CH_3_CH_2_CH_2_OH, respectively. However, the current in‐depth mechanisms for various products remain limited due to the difficulty of obtaining accurate information on key intermediates.

### 2.3 Adsorption of Intermediates

The adsorption of intermediates impacts the reaction pathways in the heterogeneous CO_2_RR process. The ideal catalyst surface should be neither strong nor weak in combination with the intermediate [57]. However, due to the same adsorption site, the adsorption energy of one intermediate is linearly scaled with that of subsequent intermediates, that is the linear scaling relationship [58]. This can be supported by calculating the adsorption energies of *CO, *COH, *CHOH, and *CHO on Ag, Au, Cu, Pd, and Pt surfaces [59]. If the adsorption of an intermediate is too strong, the desorption of products will become difficult. Obtaining a certain C_2+_ product depends on the control of each branch point in the multi‐step PCET. The linear adsorption relationship thus makes the formation of C_2+_ products much more complex than C_1_ products. Therefore, one of the major goals of CO_2_RR is to break the linear scaling relationship. According to the reduction path of Figure 3, *CO is the key intermediate for the generation of C_2+_ products, thus achieving moderate *CO adsorption is necessary to enable C−C coupling and form C_2+_ products. Constructing the dynamic coordination structure site to self‐adaptively adsorb to important intermediates has been widely studied as an effective strategy to break this linear scaling relationship [58]. Based on the FeS_1_N_3_ single‐atom catalyst, the self‐relaxation of geometric distortion and dynamic evolution of bond lengths enabled independent regulation of the *COOH and *CO intermediate adsorption energies, effectively breaking the linear scale relationship [60].

### 2.4 Performance Gap to Application

**FIGURE 4:**
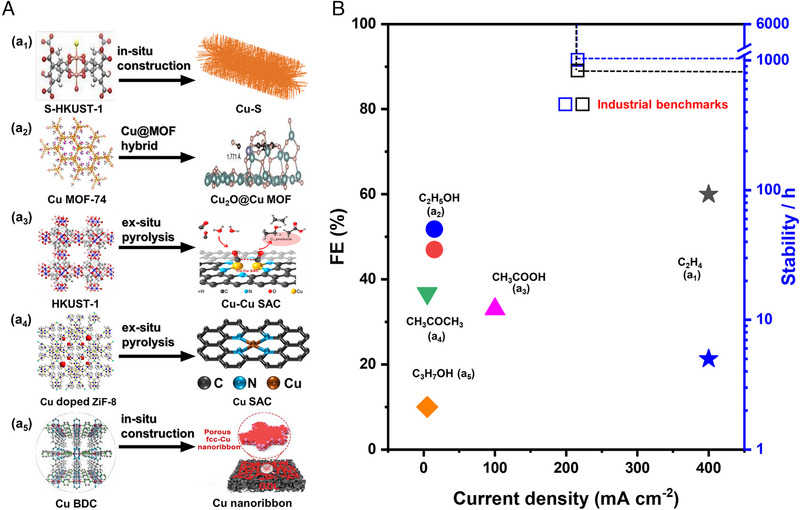
The current performance of MOF‐based Cu electrocatalysts for converting CO_2_ into typical C_2+_ products. (a_1_) Reproduced with permission [61]. Copyright 2021, Wiley. (a_2_) Reproduced with permission [65]. Copyright 2023, Wiley. (a_3_) Reproduced with permission [66]. Copyright 2021, American Chemical Society. (a_4_) Reproduced with permission [67]. Copyright 2020, Springer Nature Group. (a_5_) Reproduced with permission [68]. Copyright 2021, Wiley.

The current CO_2_‐to‐C_2+_ performance of MOF‐based Cu electrocatalysts is summarized in Figure 4. The CO_2_‐to‐C_2_H_4_ reduction has been demonstrated on a MOF(S‐HKUST‐1)‐derived Cu catalyst [61], showing a Faradaic efficiency (FE) of 60% at a current density of 400 mA cm^−2^, along with stability of 5 h. The performance of other products is even worse. As a result, these reported results are far below the level required for industrial applications (FE of 90%, current density larger than 200 mA cm^−2^, stability longer than 1000 h) [23], especially for stability (Figure 4b). For example, the positively charged Cu species (Cu^δ+^) has the inherently enhanced ability to promote the formation of the C−C bond and C_2+_ selectivity. Nevertheless, the surface Cu^δ+^ tends to be gradually reduced to Cu^0^ during the CO_2_RR process, resulting in a decline in stability. At the same time, catalyst particles tend to accumulate, resulting in a loss of catalytic surface area. In addition to the shortcomings of the catalyst itself, there are also problems in terms of the electrolyzer and the post‐separation of products. Although the classical H‐cell can screen catalysts efficiently, its application is limited by low current density and limited CO_2_ mass transfer. Flow‐cell has efficient CO_2_RR catalytic performance, yet faces stability concerns as the electrolyte will penetrate the back of the gas diffusion electrode (GDE) due to the loss of hydrophobicity over a long period of operation [62]. Also, the salt precipitation caused by the reaction between CO_2_ and OH^−^ also decreases the selectivity and stability of the system. A membrane electrode assembly (MEA)‐based electrolyzer is considered to be most promising for application, however, MEA is not suitable for liquid‐phase products as liquid‐phase products will block the small chamber space [63]. On the other hand, current electrocatalytic systems are not able to fully convert CO_2_, and the current cryogenic distillation method for gas separation consumes a lot of energy [64]. To achieve industrial application, it is necessary to develop low‐cost product separation technologies.

## 3 Design Principles of MOF‐Based Cu Catalysts Toward C_2+_ Products

In the previous section, we described the mechanisms of CO_2_ reduction, whose understanding is vital to the reasonable design of MOF‐based Cu electrocatalysts. Here, we will describe how to design an efficient electrocatalyst from three aspects: modulate the electronic properties of Cu, tune the local reaction environment, and maximize site exposure and mass transport.

### 3.1 Modulate Electronic Properties of Cu

**FIGURE 5:**
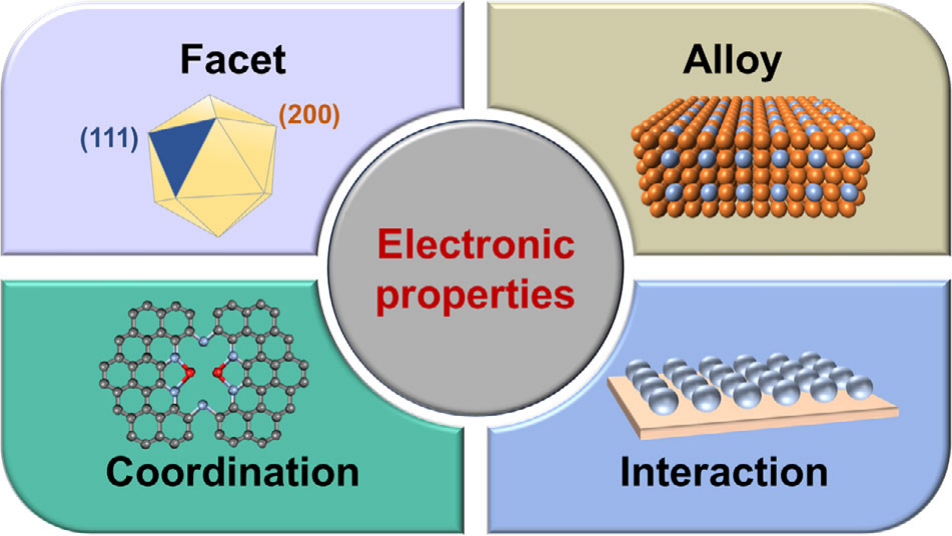
Main strategies to regulate the electronic properties of Cu.

In most cases, the relationship between catalytic performance and binding energy of intermediates on the surface of metal electrocatalysts follows Sabatier's principle [69]. Because local reactant concentrations may alter the surface coverage of the intermediate, we here emphasized “intrinsic adsorption” which is only associated with the electronic properties of catalysts. The most active catalyst should have a moderate intermediate binding strength to make the free energy close to the top of the volcano plot. The relationship between the intermediate adsorption and the electronic structure of the catalyst can be illustrated by the d‐band center theory [70, 71]. In detail, the hybridization of the metal's d‐band and the σ‐orbital of the intermediate forms the bonding and antibonding molecular orbitals. Pushing the metal's d‐band center toward the Fermi level can increase the energy level of anti‐bonding orbitals and decrease the electron occupation in anti‐bonding orbitals, thereby increasing the adsorption energy of intermediates. The purpose of tuning the electronic structure of Cu catalysts is to regulate the inherent adsorption properties of the intermediate and ideally break linear scaling relation. Next, we will discuss the main factors influencing the electronic structure of Cu catalysts, such as facet, alloy, coordination, and metal‐support interaction, as depicted in Figure 5.

#### 3.1.1 Facet

Nano‐catalysts with different crystal planes usually present various electronic properties, which further affect geometrical configurations and the adsorption ability of intermediates and in turn, influence electrocatalytic behaviors [72, 73, 74, 75]. Regarding the effects of the Cu crystal plane on pathways of CO_2_RR, it has been accepted that the Cu(111) face favors the formation of C_1_ products (such as HCOO^−^ and CH_4_); Cu(100) tends to produce C_2+_ products (such as C_2_H_4_), and other high‐energy faces can form more complex C_2+_ products [76, 77, 78, 79]. According to the DFT calculations and experimental results, certain crystal facets with various adsorption to the C_1_ or C_2_ intermediates due to the different electronic properties, determine the final products. During the formation of nanocrystals, the surface with high surface energy usually grows faster but eventually disappears. As a result, the surface of the catalyst may mostly be surrounded by the low‐energy surface, which is generally less active compared to the high‐energy surface. Through designing and modifying the structure of MOF materials, it is possible to stabilize the high‐energy surface of MOF‐derived Cu to improve the catalytic performance.

#### 3.1.2 Alloy

The formation of Cu‐based bimetallic alloys with a variety of secondary metals is a common method to alter the electronic structures of Cu. It not only benefits the formation of intermediate to C_2+_ but also promotes the C−C coupling. The catalytic properties of bimetallic catalysts for CO_2_RR vary with their surface composition and geometric structure, both of which can alter electronic properties by adjusting the d‐band position. Cao et al. [80] reported a single‐atom Bi‐modified Cu alloy catalyst with an FE(C_2+_) of 73.4%. It was found that Bi doping promotes the activation of CO_2_, accelerates the C−C coupling, and inhibits the formation of H_2_ and formate. In terms of adjusting the d‐band position of Cu, Yan et al. [81] reported that a CuCo bimetallic catalyst increased the d‐band state of Co to the Fermi level, resulting in stronger adsorption of *CO on CuCo alloy than Cu and promoting the *CO−*CO coupling to form C_2+_ products with an FE(C_2+_) of 81.3%. The proportion of metal components in alloys is also an important factor determining selectivity. Wei et al. [82] studied CO_2_ reduction on Ag_x_Cu_100‐x_ alloy. By changing the surface from Cu‐rich to Ag‐rich, the binding strength of *CO intermediate becomes weaker.

Currently, MOF‐derived alloy catalysts mainly produce C_1_ products, such as CO, HCOOH, and CH_4_ [35, 83]. The mesh‐like structure of MOF increases the distance between catalytic active sites during the alloy construction process and prevents C−C coupling. To develop MOF‐based alloy catalysts with the ability to generate C_2+_ products, the alteration of atomic arrangement and spillover effects within particles must be considered. Moreover, it should be noted that most of the previous studies are based on the experimental characterization of the prepared electrocatalysts but do not research the possible changes in the surface composition and chemical state of the electrocatalyst during the electrocatalytic process [12]. Further attention should be paid to the implementation of in situ techniques (e.g., XAFS, XPS, and liquid TEM) to obtain in‐depth information on the dynamic behavior of the CO_2_RR electrocatalysts’ structure and composition under operating conditions.

#### 3.1.3 Coordination Configuration

The local coordination configuration is a key factor in regulating the electrocatalytic performance. There is a great chance to design Cu‐based MOF with the desired site configuration for CO_2_RR. In general, it is difficult to achieve C−C coupling on a single Cu site of Cu‐MOF, while the C−C coupling can be realized by regulating the suitable distance between catalytic Cu sites. In addition, it is possible to transfer *CO from one Cu site to another site to achieve C−C coupling via designing ligands in Cu‐MOF catalysts. Furthermore, MOF has bicocentonal or tricocentonal sites with various spatial coordination configurations. Thus, there would be suitable spacing between catalytic Cu sites, where C−C coupling can be promoted. For MOF‐derived single‐atom metal catalysts, the C−C coupling is also difficult. Double‐atom catalyst is one of the main solutions to address the challenge as diatomic Cu−Cu (or other metal) sites can provide two adjacent sites to adsorb two *CO and promote C−C coupling [84]. On the other hand, regulating coordination numbers is also a productive strategy to improve the selectivity of C_2+_ products.

#### 3.1.4 Metal‐Support Interaction

The introduction of appropriate support to host metal sites can modulate the electronic structure of active sites via metal‐support interaction [85, 86]. MOF and derivatives are commonly used as porous supports in heterogeneous catalysts, which can stabilize the valence state of Cu and enhance CO_2_RR activity [87]. Zang et al. [88] reported a MOF‐derived CuO_x_@C catalyst. In the CO_2_RR process, the interaction between the carbon layer and Cu can effectively stabilize the valence state of Cu and regulate the hydrogenation path of the intermediate *HOCCH, which improves the selectivity of C_2_H_5_OH. In addition, a MOF‐derived Cu core@N_x_C core‐shell catalyst was reported by doping N in the ligand of MOF [89]. Because of the presence of Lewis base N, the catalyst has a strong affinity to acidic CO_2_, which increases the CO_2_ concentration on the surface of the catalytic site and promotes the C_2+_ product. Besides the carbon carriers, metal oxides (e.g., CeO_2_, TiO_2_, MgO, NiO, etc.) generated by calcination of metal‐based multivariate (MTV) MOF can also be used to load Cu with strong metal‐support interaction. By modulating the compositions of MTV MOF, it is expected to achieve the adsorption of different *C_1_ intermediates on dual‐sites of Cu and metal oxides, thus promoting the C−C coupling to produce C_2+_ products. In a word, the metal‐support interaction can promote C−C coupling by enhancing the adsorption of target intermediates and stabilizing the valence state of Cu in the electrocatalytic process.

### 3.2 Tune Local Reaction Environment

**FIGURE 6:**
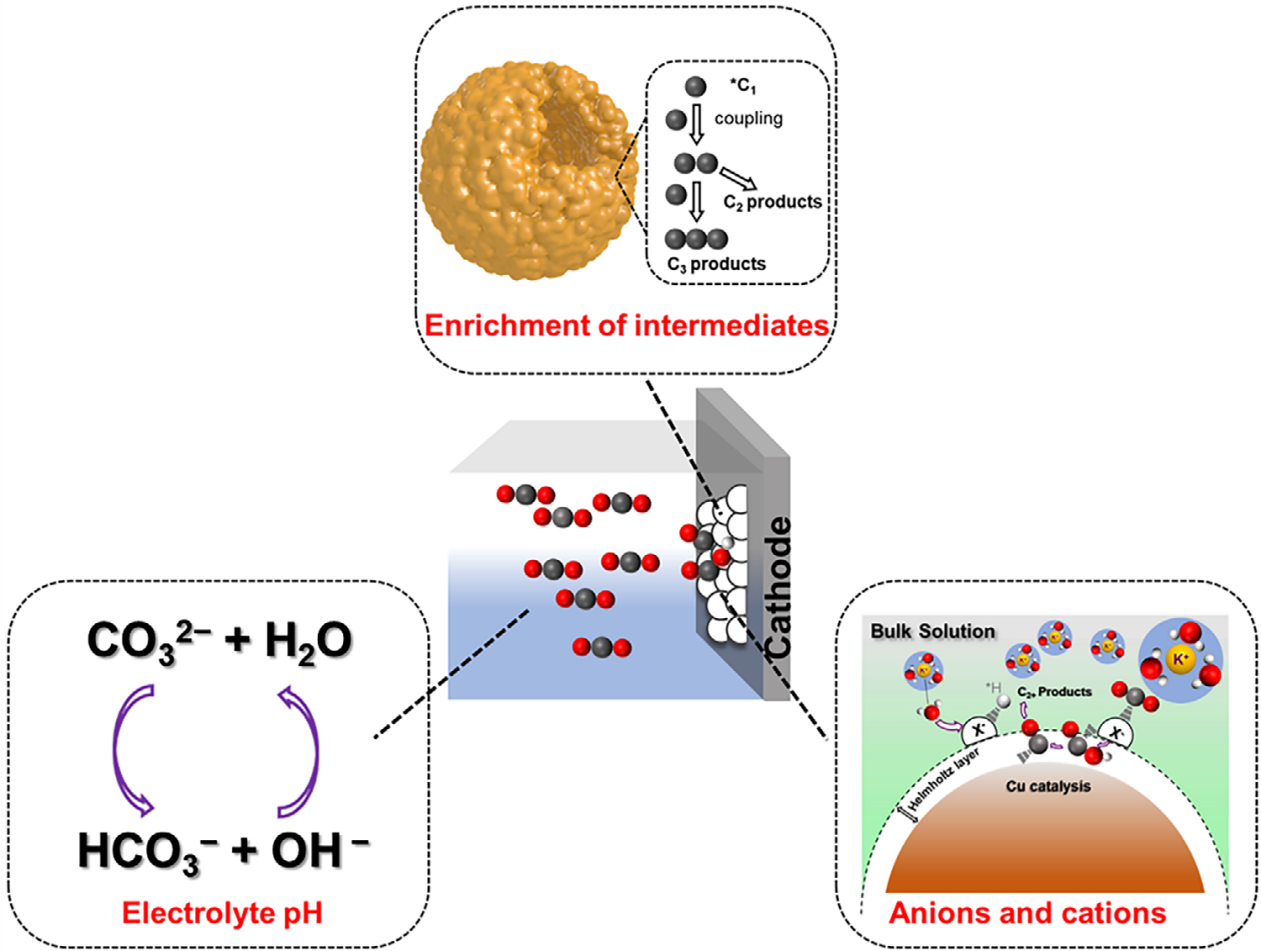
Schematic showing the local reaction environment.

Recent advances demonstrate that the selectivity and activity of CO_2_RR depend not only on the intrinsic electronic properties of the catalysts’ surface but also local reaction environment. Next, we will mainly discuss the effect of the local reaction environment on CO_2_RR from the electrolyte pH, anions and cations, and enrichment of intermediates (Figure 6).

#### 3.2.1 Electrolyte pH

In CO_2_RR, the pH near the cathode surface is more alkaline than the bulk owing to the depletion of protons by HER and CO_2_RR. The local high pH is a result of transportation constraints of proton sources from the bulk electrolyte, which is beneficial to suppressing HER. Due to the destruction of acid‐base balance, the concentration of CO_2_ in the solution also increases. However, the weak alkaline environment has a limited effect on the CO_2_ concentration on the electrocatalyst surface in practice due to the slow kinetics of CO_2_ hydrogenation. Thus, when the CO_2_RR environment becomes strongly alkaline, CO_2_ will be converted into carbonate which is not active for CO_2_RR, hampering CO_2_ mass transfer and reducing CO_2_RR activity. In sum, there needs an optimal local pH, which can effectively suppress HER and minimally affect the decrease of CO_2_RR performance [90].

In addition, it can be seen from the reaction pathway that all elemental steps of CO_2_RR are accompanied by the protonation process (Figure 3). Thus, the local pH value may determine the electrochemical steps that occur at the electrode/electrolyte interface. Particularly, under high current density (or high overpotential) conditions, the local pH tends to be stable because the rapid proton consumption at the surface leads to the depletion of dissolved CO_2_ in the boundary layer, limiting electrocatalytic performance. In addition, the electrolytes with high buffering capacity [91] and the catalyst with high hydrophobicity [92] can increase local pH because of the rapid proton depletion, being able to promote C_2_H_4_ selectivity. In sum, the local pH on the catalyst surface can regulate the product distribution over a wide potential range.

#### 3.2.2 Anions and Cations

The type of cations in the electrolyte has a great influence on the product distribution of CO_2_RR [93]. It was reported that the selectivity for C_2+_ products increases when the Stokes radius of the hydrated cation increases from Li^+^ to Cs^+^ [94]. This phenomenon is also rationalized by changes in the outer Helmholtz plane (OHP) potentially caused by differences in cation size. Specifically, smaller cations, such as Li^+^, are strongly hydrated, preventing specific adsorption of cations on the electrode surface and making CH_4_ more available. Larger cations, such as Cs^+^, are more easily adsorbed on the electrode surface, resulting in a change in OHP potential that inhibits CH_4_ formation [95, 96]. Moreover, once the cation is close to the electrode surface, it is possible to form protons by hydrolysis. This will regulate the local pH and CO_2_ concentration on the electrode surface, ultimately altering product selectivity. In addition, the cationic effect can specifically stabilize certain intermediates, thereby affecting the free energy pattern of the reduction reaction.

In addition to cations, anions should not be overlooked. At present, most studies focus on the effect of halide anion on CO_2_ reduction. Varela et al. [97] added different concentrations of KCl, KBr, and KI to 0.1 M KHCO_3_ to study the effect of Cl^−^, Br^−^, and I^−^ on the CO_2_ reduction activity and selectivity of the Cu electrocatalyst. With the addition of Cl^−^ and Br^−^, an increase in CO selectivity was observed. In the case of the addition of I^−^ to the electrolyte, a decrease in CO selectivity but an enhancement in CH_4_ production were observed. The enrichment of anions at the electrode surface is pivotal for the generation of C_2+_ products. It has been reported that Cl^−^ can be attracted to the electrode surface repeatedly and enriched by pulsed electrolysis, which facilitates *CO−*CO coupling to improve the C_2+_ product selectivity [98]. In addition, the addition of anions in the electrolyte can cause the transformation in catalyst morphology. By applying the in situ Raman spectrum, Huang et al. [99] showed that the presence of halide anions in the electrolyte modulates the coordination environment of CO binding and enhances the selectivity of Cu catalyst for C_2+_ products. The influence of the electrolyte on the selectivity of the product is also attributed to the change in the buffering capacity. Jackson et al. [100] discovered that perchlorate, sulfate, phosphate, and borate with different buffering capacities affect the selectivity of H_2_ and CH_4_. The anion with high buffering capacity acts as a hydrogen source, promoting the PCET process and inducing H_2_ and CH_4_ generation.

#### 3.2.3 Enrichment of Intermediates

The formation of C_2+_ products originates from the C−C coupling of C_1_ intermediates. Enrichment of key intermediates such as *CO, *COOH, *OCHO, *CH_2_CHO, *CH_3_CHO, and *CHO can promote their adsorption and subsequent transformation on the catalyst surface, thus advancing the C−C coupling toward the corresponding C_2+_ products. At the same time, the interaction of key intermediates with active sites can be significantly increased by enrichment of key intermediates near the catalytic site and then accelerate the CO_2_RR rate.

The micro‐physicochemical properties of catalysts have a profound effect on the enrichment of intermediates. The morphology is one of the important basic characteristics of catalysts, where the rational morphology can realize the deep contact between the intermediate and catalyst [101]. Liu et al. [102] reported a Cu catalyst with a cavity structure. The cavity structure effectively limits the flow away of *CO intermediates, thus achieving *CO enrichment and promoting C−C coupling. Similarly, Zhuang et al. [103] have also achieved the conversion of CO_2_ to C_3_ products by designing the Cu cavity structure. In addition, the two different catalysts can be combined to form a tandem catalytic system to achieve the enrichment of intermediates [78]. Detailly, most of the key intermediates (such as *CO) can be produced by the upper catalyst, which migrates to the downstream catalyst surface for further reduction. In this way, the coverage of *CO on the downstream catalyst can be greatly increased, effectively promoting the subsequent C−C coupling. For example, Wang et al. [104] designed a single‐atom Ni−N−C‐supported Cu catalyst, where Ni−N−C is used to reduce CO_2_ to CO and Cu is the site for *CO dimerization. Under this guidance, various morphology and catalyst systems can be designed to increase local C_1_ intermediates for boosted C−C coupling.

### 3.3 Site Exposure and Mass Transport

The overall performance of an electrocatalyst is determined by both the electronic properties of the active component and the number of available active sites. The former has been discussed in Section 3.1, which governs turnover frequency. The latter determines the total reaction rate, namely the turnover number, which directly reflects the overall product formation rate of CO_2_RR [105]. In practice, only the sites that can directly contact reactants/intermediates are effective catalytic sites, yet those that cannot be exposed to reactants/intermediates are inactive. Thus, the exposure of active sites and mass transport of reactive species is of great significance.

In general, there are two ways to increase the number of active sites. One is to improve the surface area; this can enhance the exposure of the active site in the entire catalyst [106]. Another is to construct a porous structure to facilitate the smooth transport of reactants/intermediates to the active site. The management of mass transport can also adjust the selectivity of the final product by controlling the local concentration of intermediates [107]. For example, if CO is not rapidly lost on the catalyst surface, it is more likely that CO will further participate in C−C coupling. Therefore, it is desirable to enhance the availability of active centers and the outflow/retention of reactants/intermediates for increasing the activity and selectivity of C_2+_ products. Based on this point, MOF‐based catalysts with high specific surface area and various porous structures would be promising platforms for enhancing electrochemically active surface area and regulating mass transport.

## 4 Preparation and Application of MOF‐Based Cu Catalysts in CO_2_‐to‐C_2+_ Reduction

Over the past few decades, a wide variety of CO_2_RR catalysts have been developed for C_2+_ generation, such as metal alloys, metal oxides, non‐metal materials, and MOF. Among these materials, MOF‐based Cu catalysts have received extensive attention because of their many advantages [108, 109, 110, 111, 112]. Firstly, the diversity of metal clusters and ligands of MOF materials can tune the adsorption ability of intermediates to meet the demand for CO_2_RR to C_2+_ products. Secondly, MOF with a network structure has a large specific surface area and high porosity, which not only increases the exposure of active sites but also facilitates the diffusion of CO_2_/intermediates to the catalytic site. In addition to pure Cu‐based MOF, MOF‐based derivatives can be prepared by ex situ high‐temperature pyrolysis and in situ electrochemical reconstruction, in which exposed facets and compositions can be readily regulated to promote the selectivity of C_2+_ products [113]. Furthermore, combining Cu with MOF to contrast Cu@MOF composites would integrate the merits of both Cu and MOF in one catalyst to promote CO_2_‐to‐C_2+_ conversion. In this section, we will introduce the design and synthesis of typical Cu‐based MOF, MOF‐derived Cu, and Cu@MOF hybrid catalysts and their application in CO_2_ reduction toward C_2+_ products.

### 4.1 Cu‐Based MOF

**FIGURE 7:**
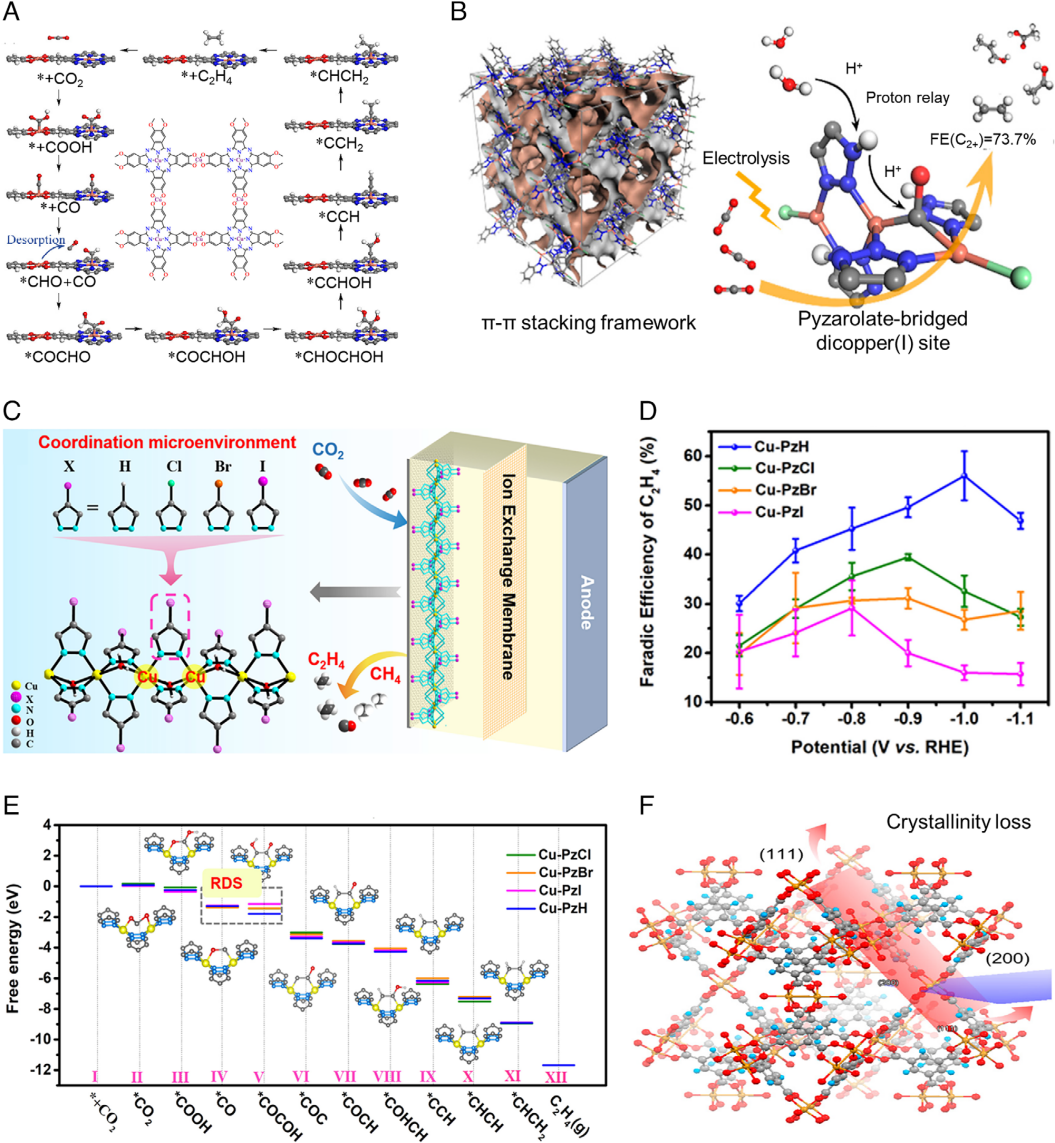
(a) Schematic diagram of C−C coupling on the PcCu‐Cu‐O. Reproduced with permission [114]. Copyright 2021, American Chemical Society. (b) Structural diagram of the CuBtz. Reproduced with permission [115]. Copyright 2022, American Chemical Society. (c) Schematic showing the effect of Cl, Br, and I on CO_2_ reduction on the [Cu(4‐XPz)_2_]_n_. (d) CO_2_RR performance for C_2_H_4_. (e) DFT calculations on various catalysts. Reproduced with permission [116]. Copyright 2021, Wiley. (f) Structural diagram of HKUST‐1 with reduced coordination number. Reproduced with permission [117]. Copyright 2018, American Chemical Society.

Cu‐based MOF has been directly used in CO_2_RR because the distance of adjacent active sites and the coordination environment can be regulated to promote the C−C coupling. Liao et al. [114] synthesized a two‐dimensional PcCu‐Cu‐O MOF, composed of (2,3,9,10,16,17,23,24‐octahydroxyphthalo‐cyaninato) copper(II) (PcCu‐(OH)_8_) ligands and the square‐planar CuO_4_ nodes (Figure 7a). The MOF exhibited good C_2_H_4_ selectivity with an FE of up to 50% because of the strong π‐π interaction between adjacent MOF layers and the high crystallinity of PcCu−Cu−O. It is noted that the distance of *CO intermediates adsorbed on the CuPc and CuO_4_ units is 8.95 Å, which is not suitable for direct *CO dimerization. It was proposed that the synergistic effect between the CuPc and CuO_4_ units plays a key role in C−C dimerization; namely, the CO produced on the CuO_4_ site can efficiently migrate and dimerize with the *CO intermediate adsorbed on the CuPc.

Because a single‐atom Cu site cannot efficiently convert CO_2_ into C_2+_ products, researchers intend to design adjacent Cu sites in MOF. Zhu et al. [115] synthesized a stable supramolecular CuBtz MOF (HBtz = benzotriazole) (Figure 7b). As a derivative of 1,2,3‐triazolates, benzotriazolates (Btz^−^) can not only exhibit pyrazolate coordination pattern to form a double copper (I) site with a pair of Cu (I) ions but also participate in the intermolecular π‐π interaction through its aromatic ring. An FE (C_2+_) of 61.6% and a current density of 1 A cm^−2^ at −1.6 V vs. RHE were achieved. DFT calculation shows that the double Cu sites are the key to enabling C−C coupling, and *CO bonded with two Cu sites is thermodynamically favorable for C−C coupling over *CO bonded with a single Cu site.

The distance of adjacent Cu active sites can be modulated by modifying the chemical structure of ligands in MOF. Wang et al. [116] synthesized a series of stable homomorphic one‐dimensional (1D) chain compounds, [Cu(4‐XPz)_2_]_n_· solvent, (X = H, Cl, Br, I; Pz = pyrazole) (Figure 7c). The distance (D_Cu‐Cu_ Cu‐PzH, Cu‐PzCl, Cu‐PzBr, and Cu‐PzI) gradually increased from 3.57 Å, 3.60 Å, and 3.61 Å to 3.63 Å, yet the dihedral Angle (*β*_Cu–Cu_) decreased from 74.48°, 72.42°, and 71.82° to 70.87°, respectively. The CO_2_RR experiment showed that the high selectivity of C_2_H_4_ (FE = 60% at −1.0 V vs. RHE) is promoted by the decrease in active site spacing (D_Cu–Cu_) and the increase in dihedral angle (*β*_Cu–Cu_) (Figure 7d). The two adjacent Cu sites can promote the *CO−*COH coupling, which has a lower potential barrier than the formation of CH_4_ (Figure 7e). Notably, C_2_H_4_ and C_2_H_5_OH are two competitive C_2_ products. Based on the reported results, *CHCOH is the key intermediate for CO_2_ reduction to C_2_H_4_ and C_2_H_5_OH, and the control in selectivity can be achieved by modulating the further hydrogenation path of *CHCOH. It has been reported that the transformation of *CHCOH to *CHCHOH generates C_2_H_5_OH, while the transformation of *CHCOH to *CCH yields C_2_H_4_ [118]. Zhao et al. [34] reported a Cu−Sn hexylaminobenzene MOF catalyst, which achieved *CO−*OCH_2_ coupling and improved the selectivity of C_2_H_5_OH rather than C_2_H_4_ due to the lower energy barrier of the subsequent hydrogenation to CH_3_−*OCH_2_ than the C—O bond fracture. In addition, the distance of adjacent Cu sites can be regulated by decreasing the coordination number of MOF to promote C−C coupling via twisting the symmetric lung‐type Cu dimers in an HKUST‐1 MOF (Figure 7f) [117]. Since different metals have different adsorption energies for intermediates, constructing MTV MOF would be a productive modification strategy to boost C−C coupling by introducing heterogeneous metals to form the double metal active sites. In the future, the rational design of the metal active sites with proper distance should take advantage of the tailorable ability of MOF to completely exert various interactions between important intermediates and different metals for C−C coupling [34, 119]. In addition, as MOF usually has poor electrochemical stability, especially under the negative potential of CO_2_, the long‐term durability of Cu‐MOF catalysts should be carefully evaluated.

### 4.2 MOF‐Derived Cu Catalysts

Although some Cu‐based MOF have been directly used as electrocatalysts for converting CO_2_ into C_2+_ products, the poor electrochemical stability under negative potentials limits their practical application under industry‐level currents [120]. Instead, converting MOF into metal, oxide, sulfide, and carbon‐based catalysts via ex situ pyrolysis in situ electrochemical reconstruction, and other methods could address the concerns. Excitedly, it is possible for MOF‐derived catalysts to retain the original advantages of MOF such as porosity and controllability [121, 122, 123].

#### 4.2.1 Cu Catalysts Derived from Ex Situ Pyrolysis of MOF

**FIGURE 8:**
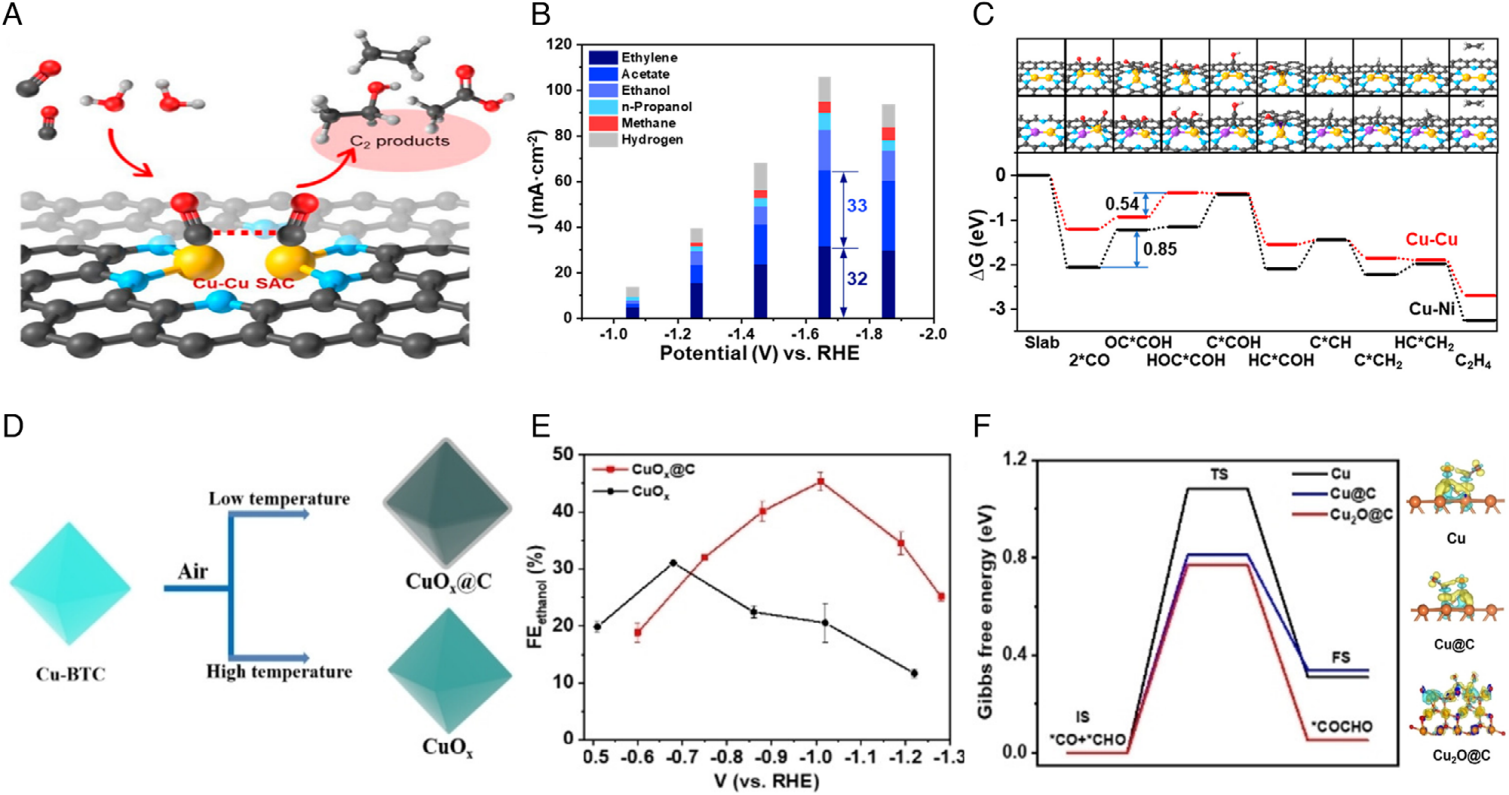
(a) Schemes of CO reduction on dual‐atom Cu−Cu sites. (b) Partial current densities on the dual Cu SAC. (c) Gibbs free energy diagram for CORR on the Cu−Cu and Cu−Ni SACs at U = 0 V. Reproduced with permission [66]. Copyright 2021, American Chemical Society. (d) Schemes showing the preparation of CuO*_x_* @C and CuO*_x_* catalysts. (e) Potential‐dependent FE of C_2_H_5_OH. (f) Energy profiles of *CO adsorption on Cu, Cu@C, and Cu_2_O@C, as well as corresponding electron density difference plots of catalysts with adsorbed *CO and *CHO. Reproduced with permission [88]. Copyright 2022, Wiley.

The ex situ pyrolysis of Cu‐MOF in an inert atmosphere can produce carbon‐supported Cu catalysts. Mononuclear MOF‐derived carbon‐supported single‐atom Cu has high atom utilization and unsaturated coordination [124]. Using Cu‐doped ZIF‐8 as a precursor, Zhao et al. [67] obtained Cu/SA‐NPC electrocatalyst under N_2_ atmosphere at 1000°C and achieved CO_2_ reduction to CH_3_COCH_3_ (FE = 36.7%). It was found that Cu−pyrrolic‐N_4_ coordination works better than Cu−pyridine‐N_4_ sites to reduce the energy barriers for CO_2_ activation and stabilize acetone‐producing intermediates. However, the generation of C_2+_ products on a single‐atom site remains difficult as the C−C coupling on one Cu site faces a high energy barrier [125]. Compared to mononuclear MOF, MOF with metal clusters can be more easily derivatized into metal particles, promising to facilitate the coupling of two *C_1_ intermediates. Zou et al. [126] reported a Cu‐BTC‐derived carbon‐supported Cu‐Ag nanoparticles catalyst for the generation of C_2_H_5_OH by *CHO−*CHO coupling at the Cu site. However, C−C coupling can only occur at the surface sites of the metal nanoparticle catalysts; resulting in low atom utilization. Dual‐site single‐atom catalysts effectively solved the above problem and gained more attention in generating C_2+_ products. Li et al. [66] developed a diatomic Cu−Cu SAC derived from HKUST‐1 (Figure 8a). The results show that two CO molecules can be adsorbed and dimerized via Cu atom pairs, resulting in an FE (C_2+_) of 91% at −1.6 V versus RHE (Figure 8b). When one of the Cu atoms is replaced by Ni, the product is mainly CH_4_ as the adsorption of *CO on Ni is too strong. DFT calculations (Figure 8c) show that the generation of HOC*COH is the RDS in the C_2_H_4_ and acetate formation, whose Gibbs free energy change on the double Cu–Cu site (0.54 eV) is lower than that on the Cu–Ni site (0.85 eV). In contrast, the free energy change of *CO hydrogenation to *CHO, the RDS for CH_4_, is 1.26 eV on the Cu–Ni site, lower than that on the Cu–Cu site (1.40 eV). In addition, MTV MOF‐derived single‐atom alloy (SAA) electrocatalysts have also been developed to promote the C–C coupling. Kim et al. [127] calcined Co‐doped Cu‐BDC MTV MOF to prepare CuCo SAA. The doping of Co accelerates the reduction of CO_2_ to CO, which improves the local *CO concentration on the Cu site to promote *CO−*CO coupling. Also, Co‐doping was found to be conducive to the conversion of *COCO‐derived *HOCCH intermediate to *CCH, improving the selectivity of C_2_H_4_ rather than C_2_H_5_OH.

Calcination in the air can convert MOF into Cu oxide. Yang et al. [128] annealed a Cu‐ASP MOF in the air to obtain porous CuO nanowires. The abundant oxide‐derived metastable Cu interface facilitates the coupling of *CO−*CO improving C_2+_ selectivity with an FE (C_2+_) of 70% in CO_2_ reduction. When the calcination process is conducted at a low temperature, the ligands of MOF will not be fully removed and will convert into carbon shells that can regulate the reaction pathway of the Cu core. For instance, Zang et al. [88] developed CuO_x_@C by pyrolysis of HKUST‐1 (Cu‐BTC) at 800°C (Figure 8d). The catalyst achieved an FE (C_2+_) up to 82 %, in which the FE of C_2_H_5_OH is 46% with a partial current density of 166 mA cm^−2^ (Figure 8e). This excellent C_2+_ selectivity contributed to the existence of Cu^+^ in CO_2_RR stabilized by carbon coating. Specifically, the carbon layer reduces the barrier and enthalpy of *CO−*CHO coupling and steers to the generation of *HOCHCH intermediate (Figure 8f), thus improving C_2_H_5_OH selectivity. The mesopore of carbon shell is expected to increase the accessibility of the active site and enrich C_1_ intermediates through the confinement effect, thus promoting the activity and selectivity of C_2_ products; however, there is still a lack of effective approaches to control the size of carbon shells during the ex situ pyrolysis of MOF. More attention should be paid to this direction in future research. The introduction of heteroatoms in the ligand of MOF can construct a functional heteroatoms‐doped carbon shell on the Cu surface. Li et al. [89] obtained a Cu@N_x_C core‐shell structure by calcining Cu‐TCNQ. The N_x_C layer helps enrich CO_2_ and *CO coverage. More importantly, HER is effectively inhibited due to the hydrophobicity of N_x_C, leading to an FE (C_2+_) of up to 80%. These works exemplify the potential of ex situ MOF‐derived materials in converting CO_2_ into C_2+_ products by adjusting compositions of MOF and pyrolysis conditions. In addition to ex situ pyrolysis, there are three other ex situ methods for converting Cu‐based MOF to metal oxides/sulfides: chemical reaction with gases/vapors, chemical reaction with solutions, and post‐chemical etching [129], which are promising directions for future research.

#### 4.2.2 Cu Catalysts Derived from In Situ Electrochemical Reconstruction of MOF

**FIGURE 9:**
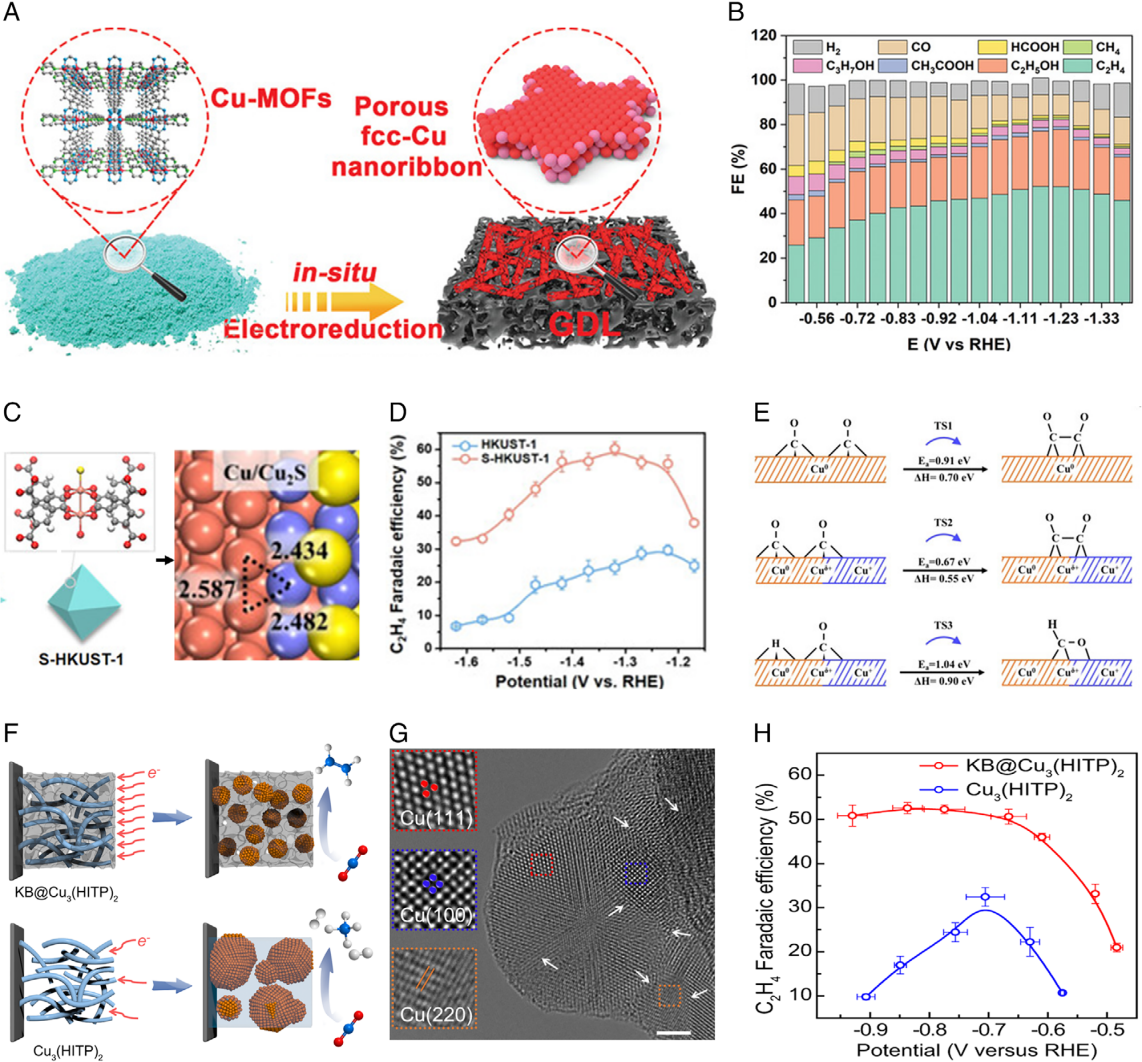
(a) Schematic illustration of the in situ electroreduction of Cu‐MOF to porous Cu nanoribbons. (b) FEs for CO_2_ reduction products on the Cu nanoribbons. Reproduced with permission [68]. Copyright 2021, Wiley. (c) Schematic showing the reconstruction of S‐HKUST‐1 into Cu/Cu_2_S. (d) FE of C_2_H_4_ for S‐HKUST‐1 and HKUST‐1 in CO_2_‐saturated 0.1 M KHCO_3_. (e) The reaction barriers and enthalpies for *CO dimerization and hydrogenation over Cu (111) and Cu/Cu_2_S surfaces. Yellow, red, gray, white, orange, and blue balls refer to S, O, C, H, Cu^0^, and Cu^δ+^ atoms, respectively. Reproduced with permission [61]. Copyright 2021, Wiley. (f) Schematic diagrams showing the modulation of CO_2_RR selectivity over the Cu_3_(HITP)_2_‐derived Cu° catalyst in the presence (top) or absence (bottom) of the carbon support. (g) TEM image of KB@Cu_3_(HITP)_2_ after CO_2_RR at −1.25 V for 10 h. (h) FEs of C_2_H_4_ at different potentials. Reproduced with permission [131]. Copyright 2021, Springer Nature Group.

For in situ derivatization, MOF precursors were subjected to electrochemical reduction in the operating CO_2_RR environment. In this process, MOF, as a pre‐catalyst, was transformed into more stable components. The resulting catalysts can be directly used for CO_2_RR [130]. MOF‐derived metals usually inherit the porous characteristics of MOF pre‐catalysts, along with better electrical conductivity. Huo et al. [68] reported the in situ electrochemical reduction of a Cu‐MOF, composed of terephthalic acid and 1, 4‐diazacycloctane ligands, to mesoporous Cu nanoribbons (Figure 9a), which can efficiently convert CO_2_ into C_2+_ with an FE reaching 82.3% and a partial current density up to 347.9 mA cm^−2^ (Figure 9b). The excellent performance contributed to the highly mesoporous structure of MOF‐derived Cu nanoribbons, which caused the enhancement in the electric field on the catalyst surface. The effect further promotes the adsorption of K^+^ and OH^−^ on the catalyst surface and reduces the thermodynamic energy barrier for the formation of *CO and subsequent *CO−*CO coupling to produce C_2+_.

Post modifications of MOF have been developed to construct new active sites during the in situ reconstruction to increase the production of C_2+_ products. Wen et al. [61] used a local sulfur doping strategy to construct isolated Cu−S motifs in a S‐HKUST‐1 MOF, which was further transformed into Cu/Cu_x_S_y_ with a rich biphasic interface via in situ electrochemical reduction (Figure 9c). The selectivity of C_2_H_4_ on the S‐HKUST‐1‐derived Cu/Cu_x_S_y_ is significantly higher than that on the S‐free HKUST‐1‐derived Cu (Figure 9d). The energy barrier for *CO hydrogenation to C_1_ products at the Cu/Cu_2_S interface was calculated to be 1.04 eV, larger than that of *CO dimerization (0.67 eV) (Figure 9e), demonstrating the more favorable C_2_ production than C_1_ at the Cu/Cu_2_S interface.

During the in situ electrochemical reconstruction of MOF, adding conductive carriers may affect the shape and size of the final catalysts. Sun et al. [131] studied CO_2_RR behaviors of Cu_3_(HITP)_2_ (HITP = 2,3,6,7,10,11‐hexylamino‐triphenyl) MOF‐derived catalysts with and without the addition of conductive Ketjen Black (KB) (Figure 9f). The role of the KB additive is to induce tiny Cu crystallites via strong KB‐Cu interaction (Figure 9g), which enhances the adsorption of *CO on grain boundary‐rich polycrystalline planes, thus promoting its coupling with *COH and reaching high C_2_H_4_ selectivity. Compared to the pristine Cu_3_(HITP)_2_, the addition of KB presents an excellent performance in a wide range of potential with FE (C_2_H_4_) > 60% (Figure 9h). Overall, via tuning MOF compositions and adding additives during in situ reconstruction, the porosity, compositions, and size of MOF‐derived Cu catalysts can be properly regulated, providing grand opportunities to suppress C_1_ and steer the CO_2_ reduction path toward C_2+_ products. The reconstruction of the active site during CO_2_ reduction commonly happens. Effective methods are highly necessary to reveal the structure‐activity relationship during the in situ reconstruction process. Typical approaches include electrochemical in situ XAS, TEM, Raman, etc. For example, Zhang et al. [132] observed the reconstruction process of HKUST‐1 with the Cu paddle wheel clusters using the in situ XAS method. The presence of Cu−O coordination confirmed the generation of oxygenated Cu clusters after CO_2_ adsorption, which facilitates the generation of C_2+_ product due to the low potential barrier for *CHOHC generation.

### 4.3 Cu@MOF Composites

**FIGURE 10:**
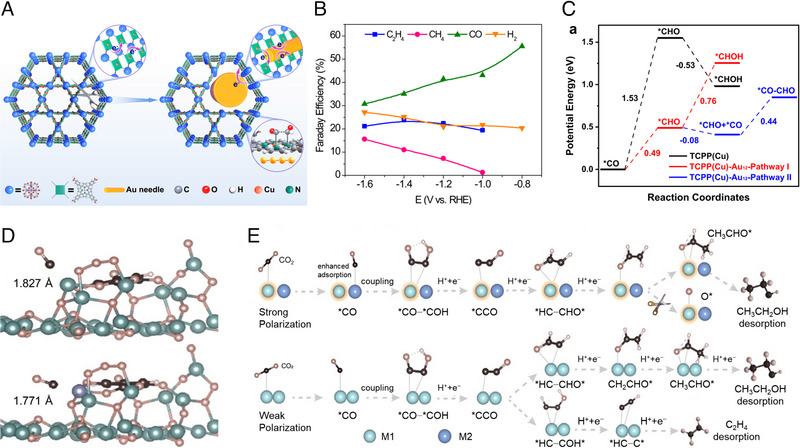
(a) Scheme showing impregnation of Au nanoneedles into PCN‐222(Cu) with cleaved ligand‐node linkage to steer the CO_2_RR pathway. (b) FEs of various products for AuNN@PCN‐222(Cu) at different potentials. (c) Free energy diagrams of the C−C coupling. Reproduced with permission [133]. Copyright 2022, Springer Nature Group. (d) The adsorption of *CO on the simplified models of Cu_2_O@NiCu‐MOF‐74/CF (upper panel) and Cu_2_O@Cu‐MOF‐74/CF (lower panel). (e) The various paths for C_2_ products on bimetallic and monometallic catalysts. Reproduced with permission [65]. Copyright 2023, Wiley.

Although many MOF have been used as precursors to yield Cu‐based catalysts, the transformation will lose some surface area or porosity of pristine MOF to some extent. In this context, the proper combination of active Cu components (e.g. Cu nanoparticles) with MOF to form Cu@MOF composite hybrids could be a promising way to modify Cu catalysts while retaining the desired properties of MOF. Xie et al. [133] implanted gold nanoneedle in a MOF‐PCN‐222 with Cu porphyrin separation center to obtain AuNN@PCN‐222 (Cu) catalyst (Figure 10a). The catalyst showed an FE (C_2_H_4_) of 52% at −1.2 V versus RHE (Figure 10b). Since the distance (10 Å) of the adjacent double Cu sites in the MOF is not suitable for C−C coupling, the generation of C_2_H_4_ was ascribed to the synergistic effect between Au and Cu‐MOF. In detail, CO_2_ reduction underwent via a tandem mechanism, where CO generated from the Au nanoneedles is reduced to *CHO on the Cu porphyrins with Au‐activated N motifs. The *CHO further combines with another CO from Au completing *CO−*CHO coupling (Figure 10c).

Using MOF as a support, the metal‐support interaction can be realized by constructing an internal plan electric field to change the C−C coupling pathway. Zhang et al. [65] constructed charge‐polarization‐enhanced asymmetric refinement structures in NiCu‐MOF‐74 nanorod arrays with encapsulated Cu_2_O (Cu_2_O@NiCu‐MOF‐74/CF). As shown in Figure 10d, the introduction of Ni effectively shortens the distance between Ni and *CO and enhances the adsorption of *CO on the other metal surface. The self‐polarized unit was constructed through this asymmetric design, which implies a stronger electron donor effect and increases the adsorption of *CO on Ni due to the different d‐band centers, which in turn increases the local *CO concentration near the Cu site and promotes *CO−*COH coupling. Moreover, the higher charge accumulation of Ni prefers to stabilize *OCHCH_3_ intermediates compared with a conventional single metal, which facilitates the generation of C_2_H_5_OH (Figure 10e). From these examples, it can be seen that combining suitable MOF with Cu can not only increase the local coverage of C_1_ intermediates to promote C−C coupling but also modify the electronic properties of Cu to control the reaction pathway toward a specific C_2+_ product of interest. Future researches are suggested to focus on how to effectively prepare integrated catalytic systems, such as the uniform and precise distribution of active Cu components into pores in MOF, and the enrichment of CO_2_ on the surface of the catalytic site can be realized by MOF to reduce the energy barrier of C−C coupling.

The typical achievements of using MOF‐based Cu catalysts for CO_2_‐to‐C_2+_ were summarized in Table 1. It shows that, though significant progress achieved, there is still a big gap between the current performance and the thresholds for industry application (e.g. FE > 90%, current density > 200 mA cm^−2^, and stability > 1000 h). On the other hand, Cu‐based MOF catalysts commonly have poor chemical and electrochemical stability during CO_2_ reduction, leading to decreased performance. Moreover, most CO_2_RR‐active MOF materials are synthesized with high cost and low yield. These drawbacks strongly suggest that future work should consider both catalytic performance and preparation costs of MOF‐based Cu catalysts.

**TABLE 1:** Summary of performance of MOF‐based Cu‐based catalysts for electrocatalytic conversion of CO_2_ to C_2+_ products.

	Catalysts	Potential (*V* vs. RHE)	Current density (mA cm^−2^)	FE C_2+_ (%)	Stability (h)	Reactor	Electrolyte	Ref.
Cu‐MOF	PcCu‐Cu‐O	−1.2	7.3	C_2_H_4_ (50)	4	H cell	0.1 M KHCO_3_	[114]
	CuBtz	−1.3	7.9	C_2_H_4_ (44)	50	H cell	0.1 M KHCO_3_	[115]
	Cu‐PzH	−1.0	346.46	C_2_H_4_ (60)	4	Flow cell	1.0 M KOH	[116]
	HKUST‐1 distorted	−1.07	262	C_2_H_4_ (45)	4	Flow cell	1.0 M KHCO_3_	[117]
MOF‐derived Cu	Cu‐SA/NPC	−0.76	≈7	CH_3_COCH_3_ (36.7)	5	H cell	0.1 M KHCO_3_	[67]
	Cu‐Cu SAC	−1.66	≈100	C_2+_ (91)	–	Flow cell	0.1 M KHCO_3_	[66]
	CoCu SAA	−1.0	550	C_2_H_4_ (45)	3	Flow cell	1.0 M KHCO_3_	[127]
	CuO nanowires	−1.3	201.4	C_2+_ (70)	12	Flow cell	0.1 M KHCO_3_	[128]
	CuO_x_@C	−1.0	315	C_2_H_5_OH (46)	56	Flow cell	1.0 M KOH	[88]
	Cu@N_x_C	−1.1	18	C_2+_ (80)	3	H cell	0.1 M KHCO_3_	[89]
	Cu nanoribbons	−1.23	200	C_2+_ (70)	10	Flow cell	1.0 M KOH	[68]
	Cu_x_S_y_	−1.35	400	C_2_H_4_ (57.2)	8	Flow cell	1.0 M KOH	[61]
	KB@Cu_3_(HITP)_2_	−1.25	22.3	C_2_H_4_ (63)	10	H cell	0.1 M KHCO_3_	[131]
Cu@MOF hybrid	AuNN@PCN‐222(Cu)	−1.2	15	C_2_H_4_ (52)	10	H cell	0.1 M KHCO_3_	[133]
	Cu_2_O@NiCu‐MOF‐74/CF	−0.61	≈25	C_2_H_5_OH (44.3)	50	H cell	0.5 M KHCO_3_	[65]

## 5 Summary and Perspectives

The electrochemical conversion of CO_2_ shows great potential in reducing atmospheric CO_2_ concentration and producing high‐value carbon products, of which C_2+_ products are more favorable than C_1_ counterparts. Designing electrocatalysts with enhanced activity, selectivity, and stability is most important in achieving the practical application of CO_2_ electrolysis. Till now, Cu is the only metal with moderate adsorption strength of *CO to realize CO_2_‐to‐C_2+_ reduction, while the performance remains limited due to the high barrier of C−C coupling and complicated reaction pathway. MOF has the advantages of high porosity, large specific surface area, and designable composition and structure, which can be used as an ideal platform to precisely design Cu catalysts. In this Review, we outlined the design principle of MOF‐based Cu catalysts for reducing CO_2_ to C_2+_ products. The regulation of electronic properties of Cu sites, local reaction environment, and site exposure and mass transport are discovered to be the main factors influencing CO_2_ reduction. Furthermore, the synthesis of Cu‐based MOF, MOF‐derived Cu, and Cu@MOF hybrid catalysts and their application in CO_2_ reduction were discussed to reveal catalytic CO_2_‐to‐C_2+_ mechanisms on MOF‐based Cu catalysts. In the following, the ongoing research directions and strategies are suggested to inspire the development of advanced MOF‐based Cu catalysts for achieving practically viable CO_2_‐to‐C_2+_ electrolysis.

1. Precise design and synthesis of high‐performance catalysts

Due to the high designability of MOF, it is an ideal platform for exploring the structure‐activity relationship between Cu catalysts and C_2+_ productions. The diversity of metal clusters and ligands in MOF can be regulated to design high‐performance MOF‐derived Cu catalysts. In addition, the regulation of metal clusters is an attractive strategy for designing asymmetric active sites and mixed metal sites, which can take advantage of the adsorption–desorption interaction of different metal sites to key intermediates or form a tandem system for C_2+_ production. Despite many design conceptions known, more general principles for the precise design of advanced MOF‐derived Cu catalysts need to be further explored. On the other hand, there remains a lack of effective methods to prepare precisely designed MOF‐based catalysts. For example, the pore structure of MOF may be changed or lost during the CO_2_ reduction or reconstruction processes, thus the final catalysts cannot fully inherit the unique advantages of MOF. On the other hand, most MOF presents poor electrochemical stability and low conductivity, which limit the application of Cu‐MOF and Cu@MOF hybrid catalysts in CO_2_ reduction. Accompanying the retention of the high activity, it is urgent to design a more stable and two‐dimensional conductive crystalline MOF for achieving long‐term durability in electrochemical reaction conditions. The main approaches to enhance the conductivity of MOF materials are (1) designing a 2D ultrathin MOF, (2) encapsulating guest molecules (e.g. highly conjugated small molecules, conductive polymers), and (3) blending them with highly conductive materials (e.g. carbon nanotube, graphene). Meanwhile, improving stability is an urgent need for MOF‐based catalyst applications, which could be achieved by: (1) strengthening coordination bonds; (2) introducing hydrophobic groups; and (3) utilizing novel chemically stable metal‐oxygen clusters as secondary building units in MOF.

2. Explore catalytic mechanisms at the solid–liquid interface

The electrochemical CO_2_RR occurs at the complex solid–liquid interface. The catalytic mechanisms depend on both the surface properties of the catalyst and the near‐electrode properties of electrolytes. The detailed experimental evidence should be explored to guide the design of effective catalysts for C_2+_ production, involving adsorption–desorption behaviors of key intermediates, optimal reaction pathways for high selectivity, and the stability of catalytic active sites under negative potentials. Therefore, it should be focused on the improvement in in situ characterization techniques (e.g. in situ XAS, FTIR, Raman, and TEM) with high spatial resolution and sensitivity to reveal the deeper catalytic mechanisms of catalysts. Regarding the impact of the local reaction environment, it is more challenging as the state of the local reaction environment is non‐static and will change as the reaction goes on. Capturing the dynamic evolution of types and concentrations of various reactive species is very difficult under operating CO_2_ reduction conditions, particularly in compact flow cell and MEA‐based electrolyzers. More advanced techniques to address the challenges are highly needed. On the other hand, the theoretical calculation is usually combined with experimental results to study the catalytic mechanism. In the DFT calculation, building the precise model that can reflect the real catalytic environment at specific active sites is highly necessary, such as the H_2_O, anions, and cations that surround the catalyst. Multiphysics‐coupled simulations are suggested to simulate the catalytic scenario in the multiscale, which would provide useful guidelines to reveal the complexity of the local reaction environment and interfacial catalytic mechanisms via combining with experimental studies.

3. Pave the way for practical application

For the practical application, the electrolyzer is difficult to meet its demand. More advanced electrolyzers should be proposed to solve salt precipitation and high internal resistance. For example, the high mass transfer efficiency of CO_2_ gas and electrolytes may be improved via novel bio/bionic designs. Currently, most MOF‐based Cu catalysts can only produce C_2+_ products in an alkaline medium. However, the reaction between CO_2_ and OH^−^ in the alkaline media can generate carbonate salts, which not only weakens the catalytic stability but also leads to significant CO_2_ loss, not viable for practical application. Using neutral and acidic media can alleviate these drawbacks, thus developing high‐performance MOF‐based Cu catalysts that can reduce CO_2_ into C_2+_ products in neutral and acidic media is highly desired. Besides, it is inevitable that minimal C_1_ products and CO_2_ mix with desired C_2+_ products. The current cryogenic distillation for gas separation suffers from high cost, and energy‐efficient separation of high‐value species such as C_2_H_4_/C_2_H_6_/C_3_H_6_/C_3_H_8_ from CO_2_ remains a great challenge. In the practical CO_2_ electrolysis, the CO_2_ reactant can be from industrial waste gases (e.g. flue gas), which commonly include impurity gases (N_2_, O_2_, H_2_S, etc.) and cause poor catalytic performance by poisoning CO_2_‐active catalysts. To solve the challenges, the input gas should be purified by adsorption‐separation and membrane separation technologies [134, 135]. Impurity gases can be diluted or removed by functionalized adsorbent and membrane to weaken the toxification of electrocatalysts with negligible CO_2_ loss during the purification process. Further, it is promising to design advanced catalytic systems to simultaneously convert CO_2_ and impure gases. For example, the C−N coupling has been realized by co‐electroreduction of CO_2_ and N_2_ to produce urea [136, 137].

The adsorption‐based gas separation over porous materials and membrane‐based gas separation technology would be promising energy‐saving methods, while the compatibility with CO_2_ electrolysis technology needs to be studied. In addition, the liquid‐phase products will dissolve in the aqueous solution, forming a salt with a lower concentration. The energy required for distillation is higher and substances with similar boiling points cannot be completely separated. Using solid‐state electrolytes would be a practically effective method to generate high‐purity liquid products for direct downstream use. The emphasis may also be focused on hybrid electro‐biosystems that could convert liquid products into energy‐rich long‐chain compounds that can be directly used in practice.

In sum, the challenges faced by Cu catalysts in CO_2_‐to‐C_2+_ reduction have been addressed to some extent by unitizing the unique properties of MOF. With in‐depth research on MOF‐based Cu catalysts, it is believed that the practical application of sustainable CO_2_‐to‐C_2+_ electrolysis can be realized in the future.

## Conflicts of Interest

The authors declare no conflicts of interest.
